# P228: Follow-up of patient colonization with multidrug-resistant microorganisms in the intensive care unit and after discharge

**DOI:** 10.1186/2047-2994-2-S1-P228

**Published:** 2013-06-20

**Authors:** QS Damaceno, AC Oliveira, J Nicoli

**Affiliations:** 1 Microbiologia, Universidade Federal de Minas Gerais, Belo Horizonte, Brazil; 2 Enfermagem Básica, Escola de Enfermagem da Universidade Federal de Minas Gerais, Belo Horizonte, Brazil

## Introduction

The screening of patients to detect colonization or infection with multidrug-resistant microorganisms, the follow-up of patient colonization, and the isolation precautions are useful tools in management of health care-associated infections (HCAIs). However, the impact evaluation for each measure control continues to require clarification.

## Objectives

To determine the epidemiological characteristics related to patient colonization with multidrug-resistant microorganisms, in the Intensive Care Unit (ICU) during the length of stay and after discharge.

## Methods

It is a prospective cohort with a follow-up of patients colonized with multidrug-resistant microorganisms during the length of stay and after discharge at two adult general ICUs from two different hospitals in Belo Horizonte, Brazil. All patients colonized or infected with resistant microorganisms, from April 2012 to January 2013, were monitored with microbiological cultures every 15 days during their length of stay at the ICU and every month after discharge until natural decolonization. Specimens were collected with a sterile swab pre-moistened with sterile saline solution. Screening were conducted by sampling the anterior nares, groin, perineum and wounds. Samples were plated on MacConkey, Cetrimide, Baird Parker agar and, Bile Esculin agar and incubated for 48 h at 37ºC. Bacteria isolated were submitted for Gram stain, catalase and Vitek (BioMérieux) tests and, for antibiogram by Bauer-Kirby method (imipenem, oxacillin, cefoxitin and ceftriaxone). For *Staphylococcus* spp the minimal inhibition concentration (MIC) of vancomycin were determined.

## Results

A total of 63 patients and four next of kin signed consent forms. The preliminary results showed the following mean time for patient decolonization with resistant microorganisms: *Pseudomonas aeruginosa* 24,4 days; *Acinetobacter baumannii* 21,1 days; *Klebsiella pneumoniae* 25 days; *Enterococcus* spp 84,5 days.

## Conclusion

The determination of mean time related to patient decolonization may favor the management of applied funds in isolating precautions, patient comfort, contributing to epidemiological surveillance in public health and others.

## Disclosure of interest

None declared

